# The Royal Naval Medical Service In War and Peace

**Published:** 1915-09-04

**Authors:** 


### THE ROYAL NAVAL MEDICAL SERVICE.

### In War and Peace.

"How can I help?" is a question that many are asking in these days. They have strength of some kind: they feel that they could add a force to the sum of the national energy if only they knew, or someone would tell them, just where to apply their strength. They know the advantage of economising strength. Nowadays many are taking a turn at tasks untried before, and whether the eager helper lends a hand at pitching sheaves in the harvest field or tries what he calls unskilled work shifting bales or packing-cases, he soon finds that there is a point where the practised hand excels him, not in muscular strength so much as in knowing just where to apply it—that is, in economy of strength. Power consists in concentrating force and directing it to the point where it best avails. How can the surgeon at this time turn his knowledge to the most account, and how best place his contribution to the nation's power? He has what many now are wishing that they had—a skill that is needed. Skill of that kind is needed at home; it is needed abroad; it is needed by civilians; it is needed by the Forces. There is none too much of it; it is precious in these days. The possessor of such skill has at his disposal a trust committed to him for the national good. He can enhance the value of the gift by placing it where it is wanted most. It is no time for considering private profit; the question is where this precious gift can be best bestowed.

That question the trustee of the gift must answer for himself. It is not the purpose of this article to plead the claim of the Naval Medical Service against other openings for the use of medical skill; but only to indicate some considerations that may influence the choice, and, if the Naval Service is chosen, to point out the way of entry.

In normal times the way of entry would be by competitive examination. It is some measure of the crisis and the need that now there are not examiners at leisure to select, even if there were a superfluity of candidates to be sifted out. The demand is for fully-qualified men, not over forty years old, and the only examination is of physical fitness. It is not to be understood that the Naval Medical Service is seriously undermanned; but normal wastage must be provided for, and no one knows when a sudden strain upon the resources of the Service may occur. What is wanted is that there shall be men on whom the authorities can quickly call; and therefore fully qualified men are asked to put down their names in readiness for the call if it should come. No immediate liability for service is incurred till the declaration has been signed, which binds the candidate to a certain six months' engagement, with a contingent liability to service for five years. Two calendar months' notice will be given of services being no longer required, and a gratuity of two months' pay will be given on discharge if the discharge is not due to misconduct or incompetency. The gratuity will be forfeited if the appointment is voluntarily resigned, which may be done if the interests of the Service allow.

In peace-time a successful candidate for admittance into the Service ranks as acting surgeon, with a pay of 14s. a day. He then enters on a two months' course at the Naval Medical School at Greenwich, where he studies tropical medicine, bacteriology, and other subjects. This is followed by a four months' course at Haslar, which includes such subjects as diving and submarine work. Not till he has passed an examination in these subjects does he become a surgeon R.N.

Obviously there is no time now for careful preparation for meeting every contingency that may arise in the course of a Service that may take a member of it to almost any part of the globe. The surgeon will be required for immediate duty, and presumably on no very distant seas. He will enter the Service therefore at once as surgeon, with a pay of 22s. a day, or £401 10s. a year. For uniform and equipment he receives an allowance of £20.

When he is attached to a ship, about 2s. a day will cover his mess expenses; if he is ashore, there are special allowances for lodging and provision.

Unmarried candidates will have the preference; but there is a scale of pensions for widows, children, and dependants of those who may be killed in action or meet their death by the acts of the enemy. In case of a surgeon being killed in action the widow receives a pension of £80, with an allowance of from £12 to £16 for boys up to the age of eighteen, and girls to twenty-one.

Fuller details and forms of application can be procured from the Director-General, Medical Department, Admiralty, S.W. After all, a first consideration should be the nation's need. For him who wants to serve, here is one point where he may apply his strength, one altar on which to consecrate his gifts to his country's use.

[*See also pages* 488 *and* 489.]

